# Pathogenic Mechanisms of *V. vulnificus* and Its Role in the Development of Sepsis

**DOI:** 10.1002/mbo3.70343

**Published:** 2026-06-22

**Authors:** Zhongying Yu, Xianzhong Zhu, Shijie Tang, Shubo Yang, Jinyu Li, Lianzhong Luo, Yan Tan, Jun Yin

**Affiliations:** ^1^ Department of Urology the 909th Hospital, School of Medicine Xiamen University Zhangzhou Fujian China; ^2^ Department of Urology General Hospital of Xizang Military Command Lhasa Tibet China; ^3^ Fujian Universities and Colleges Engineering Research Center of Marine Biopharmaceutical Resources, Xiamen Medical College Xiamen Fujian China; ^4^ Department of Pathophysiology Army Medical University Chongqing China; ^5^ Key Laboratory of Extreme Environmental Medicine, Ministry of Education of China Chongqing China; ^6^ Key Laboratory of High Altitude Medicine, PLA Chongqing China

**Keywords:** antimicrobial therapy, marine pathogens, necrotizing fasciitis, sepsis, V. vulnificus, virulence factors

## Abstract

*Vibrio vulnificus* (*V. vulnificus*) is a highly virulent marine pathogen responsible for severe infections, including necrotizing fasciitis and life‐threatening sepsis, particularly in immunocompromised individuals. This review synthesizes current knowledge on *V. vulnificus* pathogenesis, focusing on key virulence factors such as cytolysins (VvhA and MARTX), iron acquisition systems, and immune evasion strategies. The bacterium's ability to induce rapid tissue damage, endothelial dysfunction, and systemic inflammation—mediated through cytokine storms and necroptosis—underpins *V. vulnificus* high mortality rates. Risk factors include chronic liver disease, diabetes, and environmental exposure to seafood harboring *V. vulnificus* or brackish water. Clinical management hinges on early diagnosis, prompt antibiotic therapy (e.g., doxycycline combined with third‐generation cephalosporins), and surgical intervention for necrotizing infections. Despite advances, challenges persist, including antibiotic resistance and delayed diagnosis. Future directions emphasize novel therapeutics targeting virulence mechanisms, rapid diagnostics, and climate‐adaptive public health strategies to curb the rising incidence of *V. vulnificus* infections in warming coastal ecosystems. This review underscores the urgent need for multidisciplinary approaches to mitigate the global burden of this formidable pathogen.

## Graphical abstract

Sorafenib attenuates urethral scar formation by inhibiting β‐catenin signaling in fibroblasts, which downregulates the SLC7A11/GPX4 antioxidant axis and triggers ferroptosis. This β‐catenin–ferroptosis coupling effectively suppresses fibrogenesis, highlighting sorafenib as a promising drug‐repurposing candidate for urethral stricture.

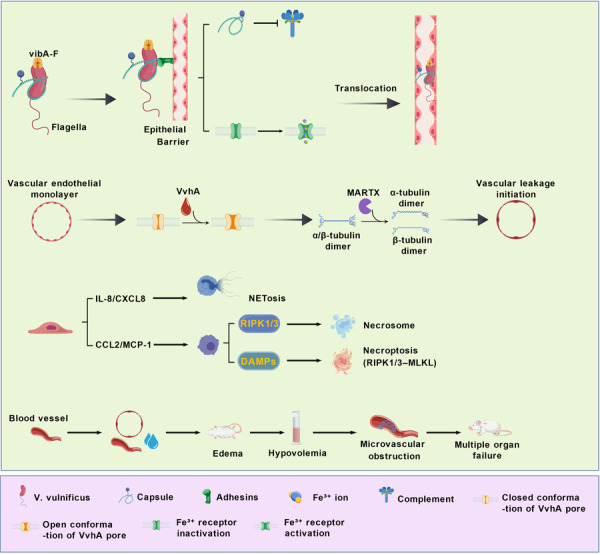

**Abbreviations**
CPS: capsular polysaccharide
DAMPs: damage‐associated molecular patterns
DIC: disseminated intravascular coagulation
LPS: lipopolysaccharide
MARTX: multifunctional autoprocessing repeats‐in‐toxin
MLKL: mixed lineage kinase domain‐like
RIPK1/3: receptor‐interacting protein kinase 1/3
SIRS: systemic inflammatory response syndrome
TCBS: thiosulfate‐citrate‐bile salts‐sucrose
VVC: V. vulnificus cytolysin
VvhA: V. vulnificus hemolysin A

## 1 Introduction

*V. vulnificus* stands as one of the most lethal marine pathogens, notorious for *V. vulnificus* rapid progression and mortality rates exceeding 50% in immunocompromised individuals (Baker‐Austin et al. 2018). While *V. vulnificus* pathogenesis involves a well‐characterized arsenal of virulence factors, a critical synthesis of the literature reveals significant complexities and unresolved questions. For instance, the relative contribution of key toxins like VvhA and MARTX appears context‐dependent, with varying importance observed between primary septicemia and wound infection models (Williams et al. 2014a). Furthermore, the role of the capsule in environmental persistence versus invasive disease remains a subject of debate (Gao et al. 2024). This review moves beyond a simple cataloging of virulence mechanisms to critically evaluate the converging and diverging evidence, highlighting knowledge gaps and proposing a unified framework for understanding the pathogenesis of *V. vulnificus* sepsis.

The epidemiology of *V. vulnificus* infections is closely tied to environmental factors, with global warming playing a pivotal role in expanding *V. vulnificus* geographic distribution. The bacterium is most prevalent in warm coastal areas, estuaries, and brackish waters, where it can persist in high concentrations during the summer months (Di et al. 2017). The primary transmission routes of *V. vulnificus* to humans are direct contact through open wounds and consumption of raw or undercooked seafood. This dual transmission pathway makes *V. vulnificus* infections difficult to control and has contributed to *V. vulnificus* increasing incidence in recent decades (Yamazaki et al. 2022).

What distinguishes *V. vulnificus* from other *Vibrio* species is *V. vulnificus* remarkable ability to cause severe, rapidly progressive infections in certain populations. The Center for Disease Control and Prevention (CDC) recognizes it as one of the three major pathogenic *Vibrio* species, alongside *Vibrio cholerae* and *Vibrio parahaemolyticus*. In the United States alone, *V. vulnificus* is responsible for approximately 80,000 illnesses and 100 deaths annually, with a mortality rate exceeding 50% in immunocompromised individuals (Candelli et al. 2025). This alarming statistic highlights the urgent need for research into *V. vulnificus* pathogenic mechanisms and effective therapeutic strategies.

While precise global figures are difficult to determine, studies in the United States suggest that *V. vulnificus* causes approximately 250 cases of illness annually, with a case fatality rate exceeding 30% (Morgado et al. 2024). In contrast, epidemiological surveillance in the Asia‐Pacific region—particularly Taiwan, South Korea, Japan, and Thailand—reports substantially higher annual incidence, often exceeding several hundred cases per year in endemic coastal provinces. In these regions, wound inoculation during aquaculture, fishing, or recreational water activities predominates over seafood consumption, and mortality frequently surpasses 50% among patients with underlying liver disease or immunosuppression (Aibinu et al. 2019). Meanwhile, European countries have historically documented only sporadic infections; however, recent warming of coastal and brackish waters has driven a marked increase in both case frequency and northward geographic expansion, with seasonal peaks now regularly observed during summer months (Elnahla et al. 2021). These regional disparities underscore important variations in primary transmission routes, high‐risk populations, and environmental drivers, highlighting the necessity of region‐specific surveillance, public health education, and clinical preparedness strategies worldwide.

While a recent review by Candelli et al provided a broad overview of *V. vulnificus* sepsis (Candelli et al. 2025), our article offers distinct value through three key innovations: (1) a proposed “Triple Threat” integrated model that synergizes toxin‐mediated damage, endothelial dysfunction, and immune dysregulation; (2) a detailed molecular elucidation of necroptosis (RIPK1/RIPK3/MLKL) as a specific amplifier of *V. vulnificus* inflammation, which was not extensively covered previously; and (3) a practical clinical management framework integrated with climate change epidemiology. By bridging molecular pathogenesis with actionable clinical and public health strategies, this review addresses critical gaps left by prior summaries and provides a unified perspective on mitigating the global burden of this formidable pathogen.

## 2 Environmental Context and Transmission Dynamics

### 2.1 Ecological Niche and Environmental Drivers

*V. vulnificus* is a halophilic bacterium that thrives in warm, saline aquatic environments, particularly coastal waters and estuaries, with optimal growth occurring at temperatures between 15°C and 40°C and salinity levels of 2%–5%. *V. vulnificus* natural reservoirs include seawater, sediments, and filter‐feeding marine organisms, where it persists as part of the indigenous microbiota. The bacterium's survival and virulence are tightly regulated by environmental cues such as temperature, salinity, pH, and nutrient availability. Notably, rising global sea surface temperatures due to climate change have expanded both the geographic range and seasonal duration of *V. vulnificus* proliferation, leading to increased incidence in previously non‐endemic regions (Candelli et al. 2025). This climate‐driven expansion poses a growing public health threat, particularly in temperate zones where summer water temperatures now regularly support bacterial growth.

### 2.2 Transmission Pathways and Regional Epidemiology

Human infections primarily occur via two routes: ingestion of contaminated seafood and direct wound exposure to contaminated water. Filter‐feeding shellfish—especially oysters, clams, and mussels—efficiently concentrate *V. vulnificus* from ambient waters, often achieving intracellular concentrations far exceeding environmental levels. Consumption of raw or undercooked shellfish, a common practice in many coastal cultures (e.g., Japan and Southeast Asia), poses a significant risk, particularly for immunocompromised individuals.

Concurrently, wound inoculation occurs when open cuts or abrasions are exposed to warm brackish or coastal water during activities such as swimming, fishing, or shellfish handling. This route is strongly associated with rapidly progressive necrotizing fasciitis and systemic sepsis.

The Asia‐Pacific region bears a substantial burden of *V. vulnificus* infections, with marked geographic variation. Taiwan reports one of the highest annual incidence rates globally (> 100 cases), often linked to wound exposure (Baker‐Austin et al. 2018). In South Korea, the pathogen is a leading cause of necrotizing soft tissue infections in coastal areas (Kim et al. 2019), while Japan sees frequent cases tied to raw seafood consumption (Aibinu et al. 2019).

### 2.3 Public Health Implications and Prevention

The interplay between environmental change, human behavior, and host susceptibility defines the transmission dynamics of *V. vulnificus*. Seasonal peaks in temperate regions (May–October in the Northern Hemisphere) contrast with year‐round transmission in tropical and subtropical zones, reflecting regional climatic conditions. Enhanced surveillance, public education on the risks of raw seafood consumption and wound exposure, and improved shellfish safety practices (e.g., depuration, post‐harvest cooling) are critical preventive measures. As climate change continues to alter marine ecosystems, adaptive public health strategies will be essential to mitigate the expanding threat of *V. vulnificus* infections. However, while environmental and behavioral factors dictate the risk of exposure, the ultimate severity of the disease is dictated by the pathogen's sophisticated molecular arsenal. Upon breaching host barriers, *V. vulnificus* deploys a complex array of virulence factors to evade immunity and inflict tissue damage, as detailed in the following section.

## 3 Virulence Factors and Pathogenic Mechanisms

The extraordinary virulence of *V. vulnificus*, characterized by a rapid progression from infection to septic shock and a mortality rate exceeding 50% (Elnahla et al. 2021), stems from a sophisticated array of pathogenic mechanisms. These include the ability to bypass host defenses through immune evasion, invade tissues via motility and enzymatic degradation, and trigger a severe inflammatory response culminating in a cytokine storm (Candelli et al. 2025). These mechanisms involve a complex interplay between bacterial virulence factors and host immune responses, ultimately leading to the devastating clinical manifestations characteristic of *V. vulnificus* infections. Understanding these pathogenic mechanisms is crucial for developing effective therapeutic strategies and preventive measures against this formidable pathogen.

### 3.1 Pore‐Forming Toxins and Cytolysins: VvhA and MARTX

Central to *V. vulnificus* pathogenicity are *V. vulnificus* diverse virulence factors, which can be broadly categorized into several functional groups. The first and perhaps most crucial group includes pore‐forming toxins and cytolysins that directly damage host cells and tissues. Among these, VvhA (hemolysin A) and MARTX (multifunctional‐autoprocessing repeats‐in‐toxin) stand out as particularly significant, though their roles are complex and context‐specific. VvhA is a potent pore‐forming toxin that oligomerizes in host cell membranes (e.g., erythrocytes, epithelial cells), creating non‐selective channels and leading to colloid‐osmotic lysis (Danielewicz et al. 2022). VvhA exerts *V. vulnificus* cytotoxic effects not only through colloid‐osmotic lysis but also by triggering intracellular signaling cascades. The formation of VvhA pores induces a rapid influx of extracellular calcium ions (Ca^2+^), which activates calmodulin‐dependent pathways and subsequently stimulates the NF‐κB and MAPK (p38, JNK) signaling pathways. This leads to the robust production of pro‐inflammatory cytokines such as TNF‐α and IL‐6, contributing to the cytokine storm observed in sepsis (Zhao et al. 2009).

Regarding MARTX, recent structural studies have elucidated the activation mechanism of this large multidomain toxin. Upon translocation into the host cytosol, MARTX binds to eukaryotic inositol hexakisphosphate (InsP6), which triggers autoproteolytic processing by *V. vulnificus* cysteine protease domain (CPD) (Jeong and Satchell 2012). This cleavage event releases multiple functionally distinct effector domains into the host cell, including the actin‐crosslinking domain (ACD) and Rho‐inactivation domain (RID). These effectors covalently modify host cytoskeletal proteins and GTPases, respectively, leading to cell rounding, loss of barrier integrity, and inhibition of phagocytic uptake (Kuo et al. 2015).

### 3.2 Iron Acquisition: A Key to Virulence

*V. vulnificus* employs sophisticated mechanisms for nutrient acquisition, particularly iron, which is essential for bacterial growth and virulence; however, in the host, iron is tightly bound to proteins such as transferrin and lactoferrin. *V. vulnificus* overcomes this restriction through high‐affinity receptors (e.g., for transferrin) and the production of the powerful siderophore vulnibactin, which chelates free iron (Kim et al. 2007). The bacterium synthesizes the catechol siderophore vulnibactin via the vib gene cluster (vibA‐F). The ferric‐vulnibactin complex is then recognized by specific outer membrane TonB‐dependent transporters (TBDTs), such as VuuA, which transport iron into the periplasmic space. Additionally, *V. vulnificus* expresses heme uptake systems (e.g., HutA and HupA receptors) that allow it to directly extract heme from host hemoglobin. This dual strategy ensures efficient iron scavenging even in the iron‐restricted environment of the human bloodstream, explaining the heightened susceptibility of patients with iron overload conditions. This efficient system allows the bacterium to scavenge iron, supporting *V. vulnificus* rapid replication. The critical role of iron acquisition is underscored by the fact that iron overload states, such as hemochromatosis, increase susceptibility to severe infection by up to 200‐fold (Okai et al. 2020). This efficient iron acquisition system allows *V. vulnificus* to scavenge iron from host tissues, supporting *V. vulnificus* rapid replication and tissue invasion. The importance of iron acquisition is further underscored by the observation that iron overload states, such as hemochromatosis, significantly increase susceptibility to severe *V. vulnificus* infections (Li and Wang 2020).

### 3.3 Immune Evasion: The Role of the Capsule

The capsular polysaccharide (CPS) is a critical determinant of *V. vulnificus* virulence, serving as a primary immune evasion factor. *V. vulnificus* presence is strongly associated with resistance to serum killing and phagocytosis, making it a dominant phenotype in clinical isolates (Liu et al. 2025a). Mechanistically, the CPS layer physically masks underlying pathogen‐associated molecular patterns (PAMPs), such as lipopolysaccharide (LPS), preventing recognition by Toll‐like receptors (TLRs). Furthermore, the capsule inhibits the activation of the complement cascade by preventing the deposition of C3b on the bacterial surface, thereby conferring resistance to serum killing. This anti‐phagocytic property allows *V. vulnificus* to survive and replicate within the bloodstream, evading clearance by neutrophils and macrophages. Although phase variation in CPS expression has been documented in vitro (Zhang et al. 2024), *V. vulnificus* in vivo significance remains an active area of research.

Despite reports of in vitro phase variation (Joseph and Wright 2004), the overwhelming prevalence of capsulated strains in clinical isolates underscores *V. vulnificus* essential role in evading host immune defenses during human infection. The CPS is a major target of the host's protective immune response, and *V. vulnificus* presence is strongly associated with resistance to serum killing and phagocytosis, making it a dominant phenotype in pathogenic contexts (Liu et al. 2025a). The in vivo significance and regulation of potential phase variation remain unclear and are an area requiring further investigation. Interestingly, *V. vulnificus* capsule expression is negatively correlated with biofilm formation (Zhang et al. 2024), reflecting a phenotypic trade‐off rather than direct inhibition by the CPS molecule itself. This inverse relationship likely represents an adaptive switch that prioritizes a planktonic, invasive state during host infection over surface‐attached environmental persistence. This suggests a complex evolutionary adaptation where the bacterium prioritizes a free‐living, planktonic state for rapid tissue invasion over biofilm formation for environmental survival.

### 3.4 Hydrolytic Enzymes: Degrading Host Barriers for Invasion

*V. vulnificus* produces a suite of hydrolytic enzymes that degrade extracellular matrix components and neutralize host defense molecules. A prominent example is VvpE, a zinc‐dependent metalloprotease. VvpE plays a critical role in tissue invasion by actively degrading host extracellular matrix components, including fibronectin, collagen, and laminin. Furthermore, VvpE can directly inactivate key host defense molecules, such as antimicrobial peptides and complement components, thereby facilitating bacterial dissemination and enhancing survival in the hostile host environment (Arunima et al. 2020).

### 3.5 Motility and Adherence: The Two‐Step Process of Colonization

The establishment of infection requires both active movement and stable attachment. Flagella are the primary organelles for motility and chemotaxis, allowing *V. vulnificus* to navigate through viscous environments like mucus and actively seek favorable niches near host tissues (Conner et al. 2016). This directed motility is a prerequisite for the subsequent step of adherence, which is mediated by dedicated surface structures. Beyond flagella, the Type IV pilus (T4P) system is crucial for initial host interaction. Specifically, the biogenesis protein PilF is essential for the assembly and function of T4P. PilF‐mediated pilus extension and retraction drive twitching motility, enabling the bacterium to crawl along host tissue surfaces and establish firm, irreversible adhesion to epithelial and endothelial cells. This PilF‐dependent adherence is a critical early step in biofilm formation on host tissues and precedes the delivery of cytotoxic effectors, particularly in wound infection models (Qin et al. 2019).

### 3.6 Regulatory Networks: Coordinating Virulence in Response to the Environment

Beyond these specific virulence factors, *V. vulnificus* exhibits complex regulatory networks that coordinate the expression of various virulence determinants in response to environmental and host signals. This ensures that virulence factors are expressed at the appropriate time and location, maximizing their effectiveness while minimizing immune detection. Key regulatory systems include.

Two‐Component Systems (TCS): These are the primary means by which *V. vulnificus* senses and responds to environmental changes such as temperature, salinity, and osmolarity. A TCS typically consists of a membrane‐bound sensor kinase and a cytoplasmic response regulator. Upon sensing a specific signal, the sensor kinase autophosphorylates and then transfers the phosphate group to the response regulator, which subsequently acts as a transcription factor to modulate the expression of target genes, including those involved in virulence (Qin et al. 2023).

The Ferric Uptake Regulator (Fur): Fur is a global regulator that plays a pivotal role in iron homeostasis. Under iron‐replete conditions, Fur binds iron and represses the expression of genes involved in iron acquisition. Conversely, under iron‐limiting conditions (such as those found in the host), Fur is inactive, allowing for the derepression and expression of siderophores and iron receptors, which are essential for survival and virulence (Pajuelo et al. 2016).

The Transcriptional Activator HlyU: In addition to global regulators, *V. vulnificus* employs specific transcriptional activators to fine‐tune virulence expression. HlyU acts as a master transcriptional activator that directly binds to the promoter regions of key virulence genes, most notably upregulating the expression of the pore‐forming toxin VvhA and the MARTX toxin gene *rtxA1*. By linking environmental stress signals to the robust expression of these cytolysins, HlyU serves as a critical nexus connecting environmental sensing to the deployment of the bacterium's offensive arsenal (Choi 2022).

The RpoS Sigma Factor: RpoS is the master regulator of the general stress response. It governs the expression of a large regulon of genes in response to various stresses, including osmotic and oxidative stress. In *V. vulnificus*, RpoS has been shown to regulate the expression of multiple virulence genes, including those involved in capsule production, toxin secretion, and oxidative stress resistance, thereby coordinating the bacterium's adaptation to the hostile host environment (Arunima et al. 2020).

Quorum Sensing (QS): *V. vulnificus* possesses QS systems that allow it to sense *V. vulnificus* own population density through the production and detection of small signaling molecules (autoinducers). While the role of QS in *V. vulnificus* virulence is still being elucidated, it is known to regulate processes such as biofilm formation and motility, which can influence the bacterium's ability to colonize and invade host tissues (Lee et al. 2022). Additionally, the σ32 heat shock response and the σ54 nitrogen regulation system are also involved in the bacterium's environmental sensing and gene regulation. This sophisticated network allows *V. vulnificus* to fine‐tune *V. vulnificus* pathogenic arsenal based on *V. vulnificus* immediate environment, whether in the host bloodstream or in estuarine waters.

### 3.7 Necroptosis: A Key Pathway Linking Toxin Activity to Inflammatory Sepsis

Recent research has highlighted the role of necroptosis, a programmed form of inflammatory cell death, in *V. vulnificus* pathogenesis. The bacterium's cytolysin VVC (*V. vulnificus* cytolysin) has been shown to directly trigger this pathway in macrophages and other immune cells (Qin et al. 2019). Necroptosis is executed through a well‐defined molecular cascade involving the activation of receptor‐interacting protein kinase 1 (RIPK1), RIPK3, and the effector protein mixed lineage kinase domain‐like (MLKL). The lytic nature of necroptosis leads to the release of intracellular contents, including damage‐associated molecular patterns (DAMPs) such as HMGB1 and ATP, which further amplify the inflammatory response by activating pattern recognition receptors on neighboring cells. The process is initiated by the activation of RIPK1, which recruits and phosphorylates RIPK3 to form the necrosome complex. Activated RIPK3 subsequently phosphorylates the pseudokinase MLKL. Phosphorylated MLKL oligomerizes and translocates to the plasma membrane, where it forms pores that disrupt membrane integrity. This lytic cell death results in the release of DAMPs, including HMGB1, ATP, and IL‐1α, which further amplify the inflammatory response and contribute to multi‐organ failure. This creates a vicious cycle of tissue damage and inflammation, significantly exacerbating the severity of sepsis. The potential of natural compounds like resveratrol to prevent *V. vulnificus*‐induced sepsis by attenuating this necroptosis pathway suggests a promising therapeutic approach (Qin et al. 2023).

The remarkable virulence of *V. vulnificus* is not solely due to individual virulence factors but rather the coordinated action of multiple factors working in concert. This multifaceted approach allows *V. vulnificus* to overcome various host barriers and defense mechanisms, leading to the severe clinical manifestations observed in infected individuals. Understanding these complex pathogenic mechanisms is essential for developing targeted therapeutic strategies that can effectively combat this formidable pathogen and prevent the devastating consequences of *V. vulnificus* infections, particularly sepsis. The major virulence factors of *V. vulnificus* and their mechanisms of action are summarized in Table 1. Collectively, these coordinated virulence mechanisms set the stage for the systemic pathological processes described in the following section. The transition from localized infection to fulminant sepsis involves a complex cascade of host‐pathogen interactions.

**Table 1:** Major virulence factors of *V. vulnificus* and their mechanisms of action.

Virulence factor	Molecular mechanism and host target	Key experimental evidence	Role in specific infection model
VvhA (Hemolysin A)	Pore‐forming toxin; oligomerizes in host cell membranes (e.g., erythrocytes, epithelial cells), causing colloid‐osmotic lysis.	In vitro hemolysis assays; crystal structure determination (Yuan et al. 2020); in vivo studies show reduced virulence in *vvhA* mutants in mouse septicemia models (Choi 2022).	Critical for initial cytotoxicity and iron acquisition from host cells. More prominent in septicemia than in wound models.
MARTX toxin	Large multifunctional toxin; delivers effector domains (e.g., Rho GTPase inactivation, actin cross‐linking) into host cytosol, disrupting cytoskeleton and inducing cell death.	Domain‐specific mutagenesis; imaging of cytoskeletal collapse in infected cells (Foulkes et al. 2019).	Essential for tissue invasion and dissemination. Plays a major role in both wound and systemic infections.
Iron acquisition systems	High‐affinity receptors (e.g., for transferrin, lactoferrin); siderophores (e.g., vulnibactin) that scavenge iron.	Growth assays under iron‐limiting conditions; in vivo studies showing hypervirulence in iron‐overloaded hosts (Lim et al. 2025).	Fundamental for survival and replication in the iron‐poor host environment. A major reason for susceptibility in patients with hemochromatosis.
CPS	O‐antigen‐like structure; confers resistance to complement‐mediated lysis (serum resistance) and phagocytosis.	In vitro serum killing assays; electron microscopy showing capsule layer; in vivo” studies showing avirulence of CPS‐deficient mutants (Eberle et al. 2020).	Primary immune evasion factor. Essential for survival in the bloodstream and a major determinant of strain virulence.
VvpE (Metalloprotease)	Degrades host extracellular matrix (fibronectin, collagen) and inactivates antimicrobial peptides/complement.	Proteolytic activity assays; in vivo tissue invasion models.	Facilitates deep tissue dissemination and immune evasion.
HlyU (Transcriptional activator)	Master regulator that directly binds promoters to upregulate expression of VvhA and rtxA1 toxins.	Promoter binding assays; virulence attenuation in hlyU mutants (Choi 2022).	Links environmental sensing to the deployment of the cytolytic arsenal.
PilF (T4P biogenesis protein)	Essential for Type IV pilus assembly, driving twitching motility and firm adhesion to host cells.	Mutagenesis studies showing loss of adhesion and twitching motility.	Critical for early‐stage colonization and biofilm formation in wound models.

## 4 The Development of Sepsis: A Multifactorial Pathological Process

### 4.1 A “Triple Threat” Model of *V. vulnificus* Sepsis Pathogenesis

**Figure 1:**
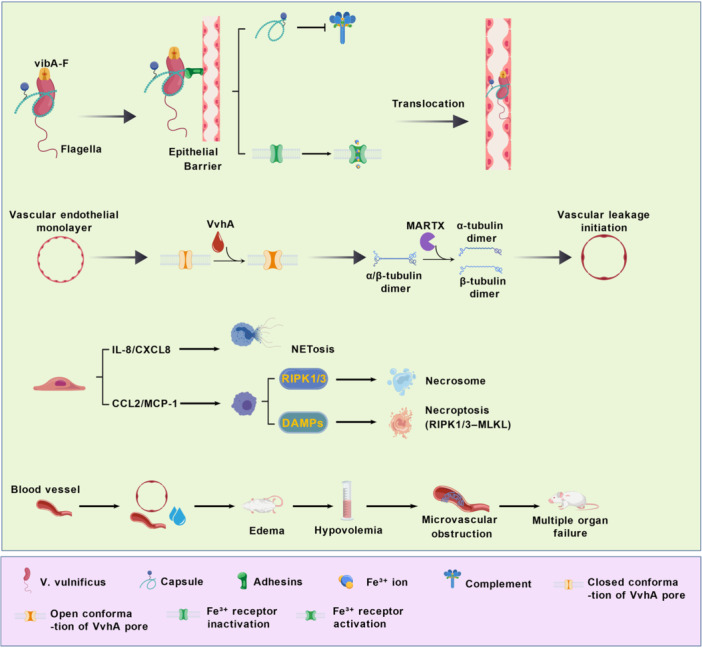
Integrated pathogenesis of *V. vulnificus* sepsis. A schematic model depicting the key steps and molecular mechanisms driving the progression from infection to sepsis, organized into four physiological tiers. (1) Invasion & adhesion: *V. vulnificus* utilizes flagella for motility and specific adhesins to bind the epithelial barrier. Concurrently, the bacterium employs the *vibA‐F* siderophore system to scavenge essential iron (Fe^3+^) from the host environment. The pathogen then facilitates translocation across the epithelial barrier into the systemic circulation. (2) Toxin‐mediated vascular damage: Within the vascular endothelial monolayer, the pore‐forming toxin VvhA induces membrane permeability (closed‐to‐open conformation), while the MARTX toxin disrupts the host cytoskeleton by cleaving α/β‐tubulin dimers. These combined actions compromise endothelial integrity, initiating vascular leakage. (3) Inflammation & cell death: Bacterial presence triggers the release of chemokines (IL‐8/CXCL8, CCL2/MCP‐1), recruiting neutrophils and monocytes/macrophages. Neutrophils undergo NETosis, releasing extracellular traps. Macrophages are induced to undergo necroptosis via the RIPK1/3‐MLKL pathway (forming the necrosome), releasing DAMPs that amplify the inflammatory cascade. (4) Sepsis & organ failure: The cumulative effects of vascular leakage result in tissue edema and systemic hypovolemia. Concurrently, microvascular obstruction (indicative of DIC) and hypoperfusion lead to multiple organ failure, illustrating the lethal synergy between direct cytotoxicity and host immune dysregulation.

To distinguish our mechanistic synthesis from existing literature, we propose an integrated “Triple Threat” schematic model (Figure 1). This framework moves beyond descriptive cataloging to illustrate how the progression from *V. vulnificus* infection to sepsis uniquely transcends the classical SIRS model through synergistic pathways. It is characterized by a “triple threat”: (1) direct, toxin‐mediated tissue destruction leading to necrotizing fasciitis and massive DAMPs release; (2) profound endothelial dysfunction and vascular leakage caused by cytolysins like VvhA and MARTX, which directly damage endothelial cells (Lee et al. 2013a); and (3) a dysregulated immune response where the initial cytokine storm (e.g., TNF‐α, IL‐6) is followed by a state of immunoparalysis in severe cases. Critically, the role of programmed necrotic cell death (necroptosis) has emerged as a key amplifier of inflammation and tissue damage, with *V. vulnificus* cytolysin VVC directly triggering this pathway in macrophages (Qin et al. 2023). This complex interplay, particularly the synergy between tissue necrosis and immune dysregulation, distinguishes *V. vulnificus* sepsis from other gram‐negative sepsis and underpins *V. vulnificus* high mortality. This integrated framework provides a more original and unified perspective on the disease process.

To ground this model in molecular genetics, we explicitly map each threat to specific *V. vulnificus* virulence determinants. The first threat is driven primarily by the VvhA hemolysin and MARTX toxin, encoded by the *vvhA* and *rtxA1* genes, which synergize to cause rapid tissue necrosis and massive DAMPs release. The second threat relies on direct endothelial targeting by these cytolysins, coupled with the *vibA–F* siderophore biosynthesis cluster and *fur* global regulator that orchestrate high‐affinity iron scavenging, fueling exponential bacterial replication in the bloodstream. The third threat is orchestrated by the *cps* operon, which directs CPS synthesis to confer serum resistance and systemic persistence, ultimately triggering a dysregulated host response. This genetic mapping transforms the ‘Triple Threat’ from a conceptual schematic into a testable, mechanistically defined pathway.

### 4.2 The Clinical Trajectory and Necroptotic Amplification of *V. vulnificus* Sepsis

Sepsis, defined by the Surviving Sepsis Campaign as life‐threatening organ dysfunction caused by a dysregulated host response, progresses with exceptional rapidity in V. vulnificus infections. Patients can advance from initial localized symptoms to fulminant septic shock and multiorgan failure within 24 to 48 h (Jin et al. 2023). This precipitous clinical deterioration is not merely a consequence of bacterial burden, but is critically amplified by the induction of necroptosis, a programmed, lytic form of cell death. As detailed in Section 3.7, V. vulnificus cytolysins (such as VVC and VvhA) directly trigger the RIPK1/RIPK3/MLKL signaling cascade in macrophages and endothelial cells (Qin et al. 2019). The lytic nature of necroptosis results in the uncontrolled release of intracellular DAMPs, including HMGB1, ATP, and IL‐1α. This massive DAMPs release acts as a potent secondary inflammatory stimulus, creating a vicious, self‐sustaining cycle of tissue damage, vascular leakage, and systemic inflammation. Consequently, the necroptotic cascade serves as the critical molecular bridge linking direct bacterial cytotoxicity to the rapid clinical trajectory of immunoparalysis and multiorgan failure, distinguishing *V. vulnificus* sepsis from other gram‐negative infections where apoptosis may dominate (Swartzendruber et al. 2022).

### 4.3 Bacterial Facilitation of Systemic Dissemination

The transition to systemic infection and sepsis is facilitated by several bacterial properties. The rapid replication rate of *V. vulnificus*, supported by efficient iron acquisition from host sources, allows for exponential bacterial growth within tissues. The production of hydrolytic enzymes that degrade extracellular matrix components facilitates tissue invasion and dissemination. Most critically, *V. vulnificus* has the remarkable ability to directly translocate across epithelial barriers, particularly in the gastrointestinal tract, entering the bloodstream and lymphatic system. This translocation is facilitated by the bacterium's motility, adherence properties, and cytolytic activities (Hayward et al. 2018).

### 4.4 Endothelial Dysfunction and Vascular Leakage

Once *V. vulnificus* translocates into the bloodstream, it initiates a catastrophic cascade of vascular pathology. The primary drivers of endothelial disruption are the pore‐forming toxin VvhA and the multifunctional MARTX toxin (Lee et al. 2013a). VvhA oligomerizes within vascular endothelial membranes to form non‐selective cation pores, while MARTX translocates into the cytosol, where autoproteolytic cleavage releases effector domains (specifically the actin‐crosslinking domain [ACD] and Rho‐inactivation domain [RID]). These effectors directly dismantle the host cytoskeleton and inactivate Rho GTPases, leading to the rapid degradation of adherens and tight junction proteins (e.g., VE‐cadherin and claudin‐5) and consequent paracellular leakage (Pérez‐Reytor et al. 2018). This direct cytotoxicity is further amplified by bacterial LPS, which engages the TLR4/MyD88/NF‐κB signaling axis in endothelial cells, promoting a pro‐adhesive phenotype (Zhao et al. 2009).

Concurrently, the host mounts a rapid innate defense that paradoxically exacerbates vascular injury. Initial recognition of *V. vulnificus* PAMPs via epithelial/endothelial TLR4/TLR5 and intracellular NLRP3 inflammasomes triggers the robust secretion of a defined pro‐inflammatory panel, including TNF‐α, IL‐1β, IL‐6, IL‐8/CXCL8, and CCL2/MCP‐1 (Qin et al. 2019). This chemokine gradient orchestrates sequential leukocyte infiltration: neutrophils are rapidly recruited and undergo excessive NETosis, releasing extracellular traps laden with histones and reactive oxygen species (ROS) that inflict severe collateral damage on the compromised endothelium (Swartzendruber et al. 2022). This is followed by the recruitment of Ly6C^+^ inflammatory monocytes and tissue‐resident macrophages (Kuo et al. 2015). However, *V. vulnificus* actively subverts this protective response: the CPS physically masks surface antigens to inhibit complement C3b deposition (Liu et al. 2025a); MARTX effectors block phagolysosomal maturation (Kwiecinski and Horswill 2020); and VvhA‐induced pore formation triggers RIPK1/RIPK3/MLKL‐mediated necroptosis in macrophages (Qin et al. 2023). This lytic cell death eliminates frontline defenders and releases additional DAMPs, converting a targeted immune response into a self‐sustaining, destructive pro‐inflammatory cascade.

### 4.5 The Cytokine Storm and Inflammatory Dysregulation

Concomitant with the vascular damage detailed in Section 4.4, V. v*ulnificus* infection triggers an intense inflammatory response that underlies the systemic nature of sepsis. The recognition of *V. vulnificus* by host pattern recognition receptors, such as TLRs, activates innate immune cells and induces the production of cytokines, chemokines, and other inflammatory mediators. The high bacterial load and potent bacterial toxins lead to excessive cytokine production, creating a cytokine storm that contributes to systemic inflammation and tissue injury. This inflammatory response, while initially aimed at controlling the infection, becomes dysregulated in sepsis, leading to widespread tissue damage and organ dysfunction

Critically, *V. vulnificus* actively subverts these host defenses to amplify the inflammatory cascade. The CPS layer physically blocks C3b deposition, neutralizing complement‐mediated opsonization and phagocytosis. MARTX effectors covalently inactivate Rho and Rac GTPases, directly impairing neutrophil chemotaxis and macrophage phagocytic uptake. Paradoxically, VvhA‐induced pore formation triggers a massive Ca^2+^ influx that hyperactivates the NF‐κB pathway in surviving immune cells. This not further amplifies the production of TNF‐α, IL‐6, and IL‐1β but also exhausts local immune resources, creating a feed‐forward loop of uncontrolled inflammation and subsequent immunoparalysis (marked by elevated IL‐10). This inflammatory response, while initially aimed at controlling the infection, becomes profoundly dysregulated in sepsis, leading to widespread tissue damage and organ dysfunction (Gutiérrez et al. 2016).

### 4.6 Sepsis‐Induced Coagulopathy and Disseminated Intravascular Coagulation (DIC)

The development of coagulation abnormalities represents another critical component of *V. vulnificus* sepsis, driven by the cytokine‐induced upregulation of tissue factor and the inflammatory cascade described previously. Sepsis‐induced coagulopathy (SIC), which frequently progresses to disseminated intravascular coagulation (DIC), is a hallmark of severe infection and a major independent driver of mortality (Dolmatova et al. 2021). The mechanisms underlying this coagulation derangement are multifactorial and intimately linked to the inflammatory cascade. First, the massive cytokine storm (particularly TNF‐α and IL‐6) induces profound upregulation of tissue factor (TF) expression on the surface of activated endothelial cells and circulating monocytes, forcefully initiating the extrinsic coagulation cascade (Liu et al. 2025b). Second, widespread endothelial damage exposes subendothelial collagen, activating the intrinsic pathway and the contact system. Concurrently, the complement system is hyperactivated (e.g., via C5a generation), which exhibits extensive cross‐talk with the coagulation cascade to further promote thrombin generation and fibrin deposition (Dolmatova et al. 2021).

This uncontrolled, systemic coagulation rapidly consumes clotting factors and platelets, leading to a hemorrhagic diathesis. Clinically, this manifests through specific coagulopathy markers that serve as strong indicators of poor prognosis in *V. vulnificus* sepsis: severe thrombocytopenia, prolonged prothrombin time (PT) and activated partial thromboplastin time (aPTT), markedly elevated d‐dimer and fibrin degradation products (FDPs), and hypofibrinogenemia (Haq and Dayal 2005). The resulting microvascular thrombosis exacerbates tissue ischemia and multiorgan hypoperfusion, creating a lethal positive feedback loop with the ongoing inflammatory response and necroptotic tissue damage.

### 4.7 Multifactorial Organ Dysfunction and Failure

Organ dysfunction in *V. vulnificus* sepsis is multifactorial in origin. Direct bacterial invasion and toxin production can damage various organs, particularly the liver, spleen, kidneys, and lungs. Vascular damage and hypotension lead to reduced organ perfusion and ischemic injury. The inflammatory response causes leukocyte sequestration and microvascular obstruction, further compromising organ function. In some cases, bacterial translocation from the gut can lead to liver and systemic inflammation. The cumulative effect of these processes is multiorgan failure, a hallmark of severe sepsis and septic shock (Xu et al. 2023).

## 5 Clinical Manifestations and Risk Factors

### 5.1 Spectrum of Clinical Syndromes and Disease Progression

*V. vulnificus* infection presents a broad clinical spectrum, ranging from self‐limited gastroenteritis to rapidly progressive sepsis with mortality rates exceeding 50%, influenced by the route of infection, bacterial virulence, and host immune status. The three principal syndromes are primary sepsis, wound infections, and gastroenteritis. Primary sepsis, typically following ingestion of contaminated seafood, manifests abruptly with fever, chills, hypotension, and altered mental status, often without a localized source, and rapidly progresses to septic shock and multiorgan failure due to bacterial translocation across the intestinal epithelium (Liu et al. 2025b). Wound infections, acquired through exposure of open wounds to warm brackish or coastal waters or contaminated seafood, often begin as cellulitis but can escalate within hours to necrotizing fasciitis—characterized by extensive tissue necrosis, hemorrhagic bullae, and systemic toxicity—earning *V. vulnificus* the colloquial designation of a “flesh‐eating bacterium” (Matsuoka et al. 2013). Gastroenteritis is generally milder, presenting with diarrhea, nausea, vomiting, and abdominal cramps due to toxin‐mediated damage to the intestinal epithelium and fluid secretion, but can progress to systemic infection in immunocompromised individuals (Li and Wang 2020). Less common atypical manifestations—including pneumonia (from aspiration), meningitis (often in cirrhotic patients), and septic arthritis—can delay diagnosis and worsen outcomes due to their non‐specific presentation (Wang et al. 2021). The hallmark of *V. vulnificus* infection is *V. vulnificus* fulminant progression, with deterioration from mild symptoms to life‐threatening illness occurring within hours, driven by potent cytolysins, immune evasion, and rapid bacterial replication (Candelli et al. 2025).

### 5.2 Diagnostic Features and Laboratory Findings

Diagnosis of *V. vulnificus* infection relies on microbiological confirmation via culture of blood, wound exudate, or stool, with the organism exhibiting characteristic green, smooth, convex colonies on thiosulfate‐citrate‐bile salts‐sucrose (TCBS) agar; however, due to the rapid clinical course, empiric treatment is often initiated before culture results are available (Jin et al. 2023). Key diagnostic clues include the pathognomonic “target sign” or “bull's eye” skin lesion—comprising a central hemorrhagic bulla surrounded by concentric zones of erythema and pallor—which is highly suggestive of *V. vulnificus* wound infection (Horseman and Surani 2011). Laboratory findings typically include leukocytosis (or leukopenia in cirrhotic patients), elevated C‐reactive protein and procalcitonin, and markers of organ dysfunction such as increased lactate, creatinine, and transaminases. The definitive diagnosis of sepsis requires evidence of infection plus organ dysfunction per Surviving Sepsis Campaign criteria, though clinical suspicion based on exposure history and characteristic signs should prompt immediate therapeutic intervention, even in the absence of microbiological confirmation (Jin et al. 2023).

### 5.3 Host, Environmental, and Prognostic Risk Factors

The progression from localized *V. vulnificus* infection to fulminant sepsis is heavily dictated by the interplay between host susceptibility, environmental exposure, and timely clinical intervention.

Host Risk Factors and Mechanistic Susceptibility: Chronic liver disease, particularly cirrhosis, represents the most significant predisposing condition, increasing the risk of severe infection by up to 200‐fold. This heightened susceptibility stems from impaired innate immune responses, splenomegaly‐mediated reduction in phagocytic clearance, and portal hypertension that facilitates bacterial translocation across the gut mucosa. Additionally, hepatic dysfunction often leads to systemic iron overload, creating a nutrient‐rich milieu that dramatically accelerates bacterial proliferation and toxin production. Other critical comorbidities include diabetes mellitus (which impairs neutrophil chemotaxis and phagocytosis), chronic alcoholism (suppressing macrophage function and disrupting epithelial barrier integrity), and immunosuppression secondary to malignancy, HIV, or corticosteroid therapy (Elnahla et al. 2021). Together, these conditions create a permissive host environment that allows *V. vulnificus* to rapidly evade early clearance and establish systemic dissemination.

Environmental and Seasonal Dynamics: Infection risk is tightly coupled to ecological and behavioral factors. *V. vulnificus* proliferation is highly temperature‐dependent, with case incidence peaking during warm months (May–October in the Northern Hemisphere) when coastal and estuarine water temperatures favor exponential bacterial growth. High‐risk exposure routes include the consumption of raw or undercooked filter‐feeding shellfish (which efficiently concentrate bacteria from ambient waters) and direct wound contact with contaminated brackish water during recreational or occupational activities (Staley et al. 2013). Furthermore, climate‐driven warming of marine ecosystems is progressively expanding the geographic range and seasonal window of *V. vulnificus*, introducing this pathogen to previously non‐endemic temperate regions and complicating public health surveillance.

Prognostic Markers and Clinical Outcomes: Clinically, patients typically present with abrupt fever, chills, and rapidly progressive cellulitis or wound infection, quickly evolving into systemic septic shock characterized by hypotension, tachycardia, oliguria, and altered mental status. Laboratory evaluation commonly reveals paradoxical leukopenia or marked leukocytosis, elevated procalcitonin and CRP, and early signs of multiorgan dysfunction (elevated transaminases, creatinine, and lactate) (Yuan et al. 2020; Mcgowan et al. 2024). Prognosis is strongly stratified by several key indicators: high APACHE II scores, severe hypoalbuminemia, pronounced coagulopathy/DIC parameters, and exaggerated early cytokine thresholds (e.g., TNF‐α, IL‐6). Mortality ranges from 50% to 60% overall (Lee et al. 2013b) but exceeds 75% in cirrhotic patients (Haq and Dayal 2005). Critically, delayed surgical debridement beyond 12–24 h is independently associated with lethal progression (Ibangha et al. 2023), whereas aggressive, multidisciplinary management initiated within 72 h of symptom onset can reduce mortality to approximately 25% (Bross et al. 2007). These data underscore that survival hinges not only on host resilience but on the rapid recognition of prognostic markers and immediate therapeutic escalation.

## 6 Diagnostic Challenges and Clinical Management

The diagnosis and management of *V. vulnificus* infections, particularly those progressing to sepsis, present significant challenges for healthcare providers. The rapid clinical progression, characteristic of *V. vulnificus* infections, necessitates a high index of suspicion and prompt intervention to improve patient outcomes. However, diagnostic delays and therapeutic challenges are common, contributing to the high mortality rates associated with this infection. This section explores the diagnostic challenges and clinical management strategies for *V. vulnificus* infections, with particular attention to those that develop into sepsis.

### 6.1 The Challenge of Rapid Clinical Progression

The primary diagnostic challenge with *V. vulnificus* infections is the rapid clinical progression, which often outpaces traditional diagnostic methods. The characteristic clinical presentation, particularly the development of bullous skin lesions in wound infections, can be pathognomonic, but recognition of these findings requires familiarity with the disease. In many cases, the clinical suspicion alone must guide initial management, as definitive microbiological confirmation may not be available for hours or days. This is particularly true for primary sepsis, where the absence of a clear portal of entry can delay recognition of *V. vulnificus* as the causative organism (Jin et al. 2023).

### 6.2 Limitations of Traditional Culture Methods

Traditional diagnostic methods for *V. vulnificus* infections include culture of blood, wound exudate, or stool, with definitive identification based on characteristic colony morphology and biochemical testing. However, these methods have significant limitations. Blood cultures, while the gold standard for diagnosing sepsis, may take 24–48 h to yield results, during which time the infection can progress from mild symptoms to life‐threatening illness. The fastidious nature of *V. vulnificus*, which requires specific growth conditions including salt supplementation, can further delay culture results if not properly identified. Wound cultures and stool cultures may provide more rapid confirmation in cases of wound infections or gastroenteritis, but again, the delay in results can impede timely intervention (Elmahdi et al. 2016).

### 6.3 Molecular Diagnostics: Rapid Identification for Timely Intervention

Recent advances in molecular diagnostics offer a transformative solution to the time‐consuming nature of traditional culture methods. Polymerase chain reaction (PCR) and *V. vulnificus* variants can detect *V. vulnificus* DNA directly from clinical specimens (blood, wound exudate) within hours, enabling rapid diagnosis and guiding prompt therapeutic decisions.

The most commonly targeted genes for PCR‐based detection are the *vvhA* gene, which encodes the species‐specific hemolysin/cytolysin, and the 16S rRNA gene, a highly conserved bacterial marker. Assays targeting the *vvhA* gene are particularly valuable due to their high specificity for *V. vulnificus*. Numerous studies have validated the performance of these PCR assays, demonstrating high sensitivity (often > 95%) and specificity (approaching 100%) in clinical settings (Trinh et al. 2017).

Next‐generation sequencing (NGS) and emerging technologies like loop‐mediated isothermal amplification (LAMP) represent even more rapid and potentially point‐of‐care approaches for the future. These methods hold promise for further reducing diagnostic delays and improving the management of *V. vulnificus* sepsis. While current management strategies provide a foundation, addressing the remaining gaps in diagnostics, therapeutics, and prevention requires innovative research directions as outlined below.

## 7 Future Directions and Research Challenges

### 7.1 Systems Biology Approaches to Host‐Pathogen Interactions

A fundamental priority is to fully elucidate the molecular mechanisms driving sepsis using systems biology. Advanced omics technologies (transcriptomics, proteomics, metabolomics) should be integrated to map bacterial virulence programs and host responses simultaneously. This holistic approach will move beyond descriptive cataloging toward predictive models of *V. vulnificus*‐induced sepsis, identifying critical nodes for intervention (Mifflin et al. 2020).

### 7.2 Novel Therapeutics: Anti‐Virulence Strategies

Targeting virulence factors offers a promising alternative to traditional antibiotics. Anti‐virulence therapies, such as inhibitors of MARTX autoprocessing or CPS biosynthesis, could disarm the pathogen without exerting selective pressure for resistance. Additionally, host‐directed therapies (e.g., necroptosis inhibitors like resveratrol) may mitigate the cytokine storm and improve survival in high‐risk populations (Pajuelo et al. 2016; Lydon et al. 2021).

### 7.3 Addressing Antibiotic Resistance and Developing Alternative Antimicrobial Strategies

Although *V. vulnificus* remains largely susceptible to current first‐line regimens, the documented emergence of antibiotic resistance in related *Vibrio* species raises concerns about future treatment efficacy (Elmahdi et al. 2016). Research into novel antimicrobial agents is therefore essential, including next‐generation small molecules, antimicrobial peptides (which target bacterial membranes with lower resistance risk), and bacteriophage therapy. Additionally, photodynamic antimicrobial chemotherapy (PACT) using blue light has shown promising results, as *V. vulnificus* exhibits high susceptibility to ROS generated by photosensitization. This modality offers advantages such as broad‐spectrum activity, minimal resistance development, and suitability for topical use in wound infections, warranting further optimization and clinical validation (Mala et al. 2014).

### 7.4 Rapid Diagnostics and Vaccine Development

The rapid progression of infection demands point‐of‐care diagnostics. Next‐generation molecular assays, including LAMP and CRISPR‐based detection systems, can deliver results within hours, significantly reducing diagnostic delays. Complementing diagnostics, vaccine development using recombinant cytolysins, inactivated whole cells, or mRNA platforms could reduce disease burden. Passive immunization with hyperimmune globulins may also offer short‐term protection during peak transmission seasons (Lee et al. 2014).

### 7.5 Integrating Climate Science, Surgical Optimization, and Clinical Guidelines

Unlike previous reviews that treat epidemiology and clinical management separately, we integrate climate change projections directly with clinical guideline optimization. Climate change is expanding the geographic and seasonal range of *V. vulnificus* due to rising sea temperatures and altered coastal ecosystems, necessitating predictive ecological models to forecast outbreaks and guide public health interventions in vulnerable regions (Brumfield et al. 2023). Concurrently, clinical research must focus on optimizing surgical management for necrotizing fasciitis, including studies comparing the timing and extent of debridement, the role of adjunctive therapies (e.g., hyperbaric oxygen, negative pressure wound therapy), and individualized decision‐making based on host and infection factors (Wu et al. 2022). Finally, the development of standardized, evidence‐based treatment guidelines—derived from systematic reviews and multicenter studies—could reduce variability in care and improve survival outcomes (Williams et al. 2014b). Exploring innovative wound care, including bioengineered skin substitutes and regenerative therapies, may further preserve limb function and reduce morbidity (Jeong and Satchell 2012). Together, these multidisciplinary efforts—from molecular science to public health—are essential to combat the rising threat of *V. vulnificus* sepsis.

## 8 Conclusion

*V. vulnificus* exemplifies a pathogen whose exceptional lethality arises from the synergistic integration of multiple virulence systems. *V. vulnificus* ability to rapidly acquire iron, evade immune clearance via *V. vulnificus* capsule, and inflict direct tissue damage through potent cytolysins creates a perfect storm for the development of fulminant sepsis. While significant progress has been made, critical gaps remain, particularly in understanding the in vivo regulation of virulence and the precise contribution of necroptosis to disease progression. Future research must move beyond descriptive cataloging and embrace a systems‐level approach to unravel the complex host‐pathogen dynamics of this formidable bacterium.

## Author Contributions

**Zhongying Yu:** conceptualization, funding acquisition, writing – original draft. **Xianzhong Zhu:** conceptualization. **Shijie Tang:** conceptualization. **Shubo Yang:** conceptualization. **Jinyu Li:** conceptualization, funding acquisition. **Lianzhong Luo:** writing – review and editing. **Yan Tan:** writing – review and editing, writing – original draft. **Jun Yin:** writing – review and editing, writing – original draft.

## Ethics Statement

The authors have nothing to report.

## Consent

The authors have nothing to report.

## Conflicts of Interest

The authors declare no conflicts of interest.

## Data Availability

No third‐party data were used in this study. All data discussed are derived from previously published literature as cited in the reference list. Data sharing not applicable to this article as no datasets were generated or analyzed during the current study.

## References

[mbo370343-bib-0001] Aibinu, I. E. , P. M. Smooker , and A. L. Lopata . 2019. “Anisakis Nematodes in Fish and Shellfish‐ From Infection to Allergies.” International Journal for Parasitology: Parasites and Wildlife 9: 384–393. 31338296 10.1016/j.ijppaw.2019.04.007 PMC6626974

[mbo370343-bib-0002] Arunima, A. , S. K. Swain , S. Ray , B. K. Prusty , and M. Suar . 2020. “RpoS‐regulatedSEN1538gene Promotes Resistance to Stress and Influencessalmonella Entericaserovar Enteritidis Virulence.” Virulence 11, no. 1: 295–314. 32193977 10.1080/21505594.2020.1743540 PMC7161692

[mbo370343-bib-0004] Baker‐Austin, C. , J. D. Oliver , M. Alam , et al. 2018. “Vibrio Spp. Infections.” Nature Reviews Disease Primers 4: 1–19. 10.1038/s41572-018-0005-8 30002421

[mbo370343-bib-0005] Bross, M. H. , K. Soch , R. Morales , and R. B. Mitchell . 2007. “Vibrio Vulnificus Infection: Diagnosis and Treatment.” American Family Physician 76, no. 4: 539–544. 17853628

[mbo370343-bib-0006] Brumfield, K. D. , A. J. Chen , M. Gangwar , et al. 2023. “Environmental Factors Influencing Occurrence of Vibrio Parahaemolyticus and Vibrio vulnificus.” Applied and Environmental Microbiology 89, no. 6: e00307‐23. 37222620 10.1128/aem.00307-23 PMC10304686

[mbo370343-bib-0007] Candelli, M. , M. Sacco Fernandez , C. Triunfo , et al. 2025. “Vibrio Vulnificus—A Review With a Special Focus on Sepsis.” Microorganisms 13, no. 1: 128. 39858896 10.3390/microorganisms13010128 PMC11768060

[mbo370343-bib-0008] Choi, S. H. 2022. “A Small‐Molecule Inhibitor VM17 Targeting a Transcriptional Regulator HlyU Attenuates the Virulence of Vibrio Vulnificus.” FASEB Journal 36, no. S1: 00R70.

[mbo370343-bib-0009] Conner, J. G. , J. K. Teschler , C. J. Jones , and F. H. Yildiz . 2016. “Staying Alive: Vibrio Cholerae's Cycle of Environmental Survival, Transmission, and Dissemination.” Microbiology Spectrum 4, no. 2: VMBF‐0015‐2015. 10.1128/microbiolspec.VMBF-0015-2015 PMC4888910 27227302

[mbo370343-bib-0010] Danielewicz, N. , F. Rosato , W. Dai , W. Römer , W. B. Turnbull , and J. Mairhofer . 2022. “Microbial Carbohydrate‐Binding Toxins—From Etiology to Biotechnological Application.” Biotechnology Advances 59: 107951. 35398203 10.1016/j.biotechadv.2022.107951

[mbo370343-bib-0011] Di, D. Y. W. , A. Lee , J. Jang , D. Han , and H. G. Hur . 2017. “Season‐Specific Occurrence of Potentially Pathogenic Vibrio Spp. on the Southern Coast of South Korea.” Applied and Environmental Microbiology 83, no. 3: e02680‐16. 27836844 10.1128/AEM.02680-16 PMC5244290

[mbo370343-bib-0012] Dolmatova, E. V. , K. Wang , R. Mandavilli , and K. K. Griendling . 2021. “The Effects of Sepsis on Endothelium and Clinical Implications.” Cardiovascular Research 117, no. 1: 60–73. 32215570 10.1093/cvr/cvaa070 PMC7810126

[mbo370343-bib-0013] Eberle, K. C. , S. J. Hau , S. L. Luan , et al. 2020. “Generation and Evaluation of a Glaesserella (Haemophilus) parasuis Capsular Mutant.” Infection and Immunity 88, no. 5: e00879‐19. 32094250 10.1128/IAI.00879-19 PMC7171231

[mbo370343-bib-0014] Elmahdi, S. , L. V. DaSilva , and S. Parveen . 2016. “Antibiotic Resistance of Vibrio Parahaemolyticus and Vibrio vulnificus in Various Countries: A Review.” Food Microbiology 57: 128–134. 27052711 10.1016/j.fm.2016.02.008

[mbo370343-bib-0015] Elnahla, A. , A. S. Attia , E. Toraih , et al. 2021. “Prognostic Factors of Mortality in Vibrio Vulnificussepsis and Soft Tissue Infections: Meta‐Analysis.” Surgical Infections 22, no. 9: 928–939. 33970025 10.1089/sur.2020.243

[mbo370343-bib-0017] Foulkes, D. M. , K. McLean , A. S. Haneef , et al. 2019. “Pseudomonas Aeruginosa Toxin ExoU as a Therapeutic Target in the Treatment of Bacterial Infections.” Microorganisms 7, no. 12: 707. 31888268 10.3390/microorganisms7120707 PMC6955817

[mbo370343-bib-0018] Gao, S. , W. Jin , Y. Quan , et al. 2024. “Bacterial Capsules: Occurrence, Mechanism, and Function.” NPJ Biofilms and Microbiomes 10, no. 1: 21. 38480745 10.1038/s41522-024-00497-6 PMC10937973

[mbo370343-bib-0019] Gutiérrez, J. , T. Escalante , A. Rucavado , C. Herrera , and J. Fox . 2016. “A Comprehensive View of the Structural and Functional Alterations of Extracellular Matrix by Snake Venom Metalloproteinases (SVMPs): Novel Perspectives on the Pathophysiology of Envenoming.” Toxins 8, no. 10: 304. 27782073 10.3390/toxins8100304 PMC5086664

[mbo370343-bib-0022] Haq, S. M. , and H. H. Dayal . 2005. “Chronic Liver Disease and Consumption of Raw Oysters: A Potentially Lethal Combination—A Review of Vibrio vulnificus Septicemia.” American Journal of Gastroenterology 100, no. 5: 1195–1199. 15842598 10.1111/j.1572-0241.2005.40814.x

[mbo370343-bib-0023] Hayward, J. A. , A. Mathur , C. Ngo , and S. M. Man . 2018. “Cytosolic Recognition of Microbes and Pathogens: Inflammasomes in Action.” Microbiology and Molecular Biology Reviews 82, no. 4: e00015‐18. 30209070 10.1128/MMBR.00015-18 PMC6298609

[mbo370343-bib-0025] Horseman, M. A. , and S. Surani . 2011. “A Comprehensive Review of Vibrio Vulnificus: An Important Cause of Severe Sepsis and Skin and Soft‐Tissue Infection.” International Journal of Infectious Diseases 15, no. 3: e157–e166. 21177133 10.1016/j.ijid.2010.11.003

[mbo370343-bib-0026] Ibangha, I. A. I. , D. C. Digwo , C. A. Ozochi , M. C. Enebe , C. N. Ateba , and V. N. Chigor . 2023. “A Meta‐Analysis on the Distribution of Pathogenic Vibrio Species in Water Sources and Wastewater in Africa.” Science of the Total Environment 881: 163332. 37028683 10.1016/j.scitotenv.2023.163332

[mbo370343-bib-0027] Jeong, H. G. , and K. J. F. Satchell . 2012. “Additive Function of MARTX(Vv) and VvhA Cytolysins Promotes Rapid Growth and Epithelial Tissue Necrosis During Intestinal Infection.” PLoS Pathogens 8, no. 3: e1002581. 22457618 10.1371/journal.ppat.1002581 PMC3310748

[mbo370343-bib-0028] Jin, L. , W. Liao , M. Jiang , et al. 2023. “A Case Report of Vibrio Vulnificus Sepsis in a Diabetic Patient.” Heliyon 9, no. 6: e16521. 37251457 10.1016/j.heliyon.2023.e16521 PMC10220368

[mbo370343-bib-0029] Joseph, L. A. , and A. C. Wright . 2004. “Expression of Vibrio Vulnificuscapsular Polysaccharide Inhibits Biofilm Formation.” Journal of Bacteriology 186, no. 3: 889–893. 14729720 10.1128/JB.186.3.889-893.2004 PMC321485

[mbo370343-bib-0030] Kim, C. M. , R. Y. Park , M. H. Choi , H. Y. Sun , and S. H. Shin . 2007. “Ferrophilic Characteristics of Vibrio Vulnificusand Potential Usefulness of Iron Chelation Therapy.” Journal of Infectious Diseases 195, no. 1: 90–98. 17152012 10.1086/509822

[mbo370343-bib-0031] Kim, S. E. , H. K. Kim , S. M. Choi , et al. 2019. “In Vitro Synergy Andin Vivoactivity of Tigecycline‐Ciprofloxacin Combination Therapy Against Vibrio vulnificus Sepsis.” Antimicrobial Agents and Chemotherapy 63, no. 10: e00310‐19. 31332060 10.1128/AAC.00310-19 PMC6761547

[mbo370343-bib-0032] Kuo, S. Y. , M. C. Chou , S. L. Lee , et al. 2015. “Vibrio vulnificus RtxA1 Modulated Calcium Flux Contributes Reduced Internalization in Phagocytes.” Life Sciences 132: 55–60. 25916802 10.1016/j.lfs.2015.03.027

[mbo370343-bib-0033] Kwiecinski, J. M. , and A. R. Horswill . 2020. “Staphylococcus aureus Bloodstream Infections: Pathogenesis and Regulatory Mechanisms.” Current Opinion in Microbiology 53: 51–60. 32172183 10.1016/j.mib.2020.02.005 PMC7244392

[mbo370343-bib-0034] Lee, C. T. , D. Pajuelo , A. Llorens , et al. 2013a. “MARTX of Vibrio Vulnificus Biotype 2 Is a Virulence and Survival Factor.” Environmental Microbiology 15, no. 2: 419–432. 22943291 10.1111/j.1462-2920.2012.02854.x

[mbo370343-bib-0035] Lee, K. W. , Y. Wen , N. Y. Park , and K. S. Kim . 2022. “Quorum Sensing and Iron‐Dependent Coordinated Control of Autoinducer‐2 Production via Small RNA RyhB In.” Scientific Reports 12, no. 1: 831. 35039556 10.1038/s41598-021-04757-9 PMC8764119

[mbo370343-bib-0036] Lee, S. H. , B. H. Chung , and W. C. Lee . 2013b. “Retrospective Analysis of Epidemiological Aspects of *Vibrio Vulnificus* Infections in Korea in 2001–2010.” Japanese Journal of Infectious Diseases 66, no. 4: 331–333. 23883847 10.7883/yoken.66.331

[mbo370343-bib-0037] Lee, T. H. , M. H. Kim , C. S. Lee , J. H. Lee , J. H. Rhee , and K. M. Chung . 2014. “Protection Against Vibrio Vulnificus Infection by Active and Passive Immunization With the C‐Terminal Region of the RtxA1/MARTXVv Protein.” Vaccine 32, no. 2: 271–276. 24252692 10.1016/j.vaccine.2013.11.019

[mbo370343-bib-0038] Li, G. , and M. Y. Wang . 2020. “The Role of Vibrio Vulnificus Virulence Factors and Regulators in Its Infection‐Induced Sepsis.” Folia Microbiologica 65, no. 2: 265–274. 31840198 10.1007/s12223-019-00763-7

[mbo370343-bib-0039] Lim, C. , C.‐Y. Zhang , G. Cheam , et al. 2025. “Essentiality of the Virulence Plasmid‐Encoded Factors in Disease Pathogenesis of the Major Lineage of Hypervirulent Varies in Different Infection Niches.” EBioMedicine 115: 105683. 40184910 10.1016/j.ebiom.2025.105683 PMC12002934

[mbo370343-bib-0040] Liu, L. , T. Zhang , L. Li , et al. 2025b. “Machine Learning and Bioinformatics to Identify Coagulation Biomarkers in Sepsis‐Related Kidney Injury.” Shock 64, no. 1: 130–137. 40208026 10.1097/SHK.0000000000002600

[mbo370343-bib-0041] Liu, X. , Q. Xu , X. Yang , et al. 2025a. “Capsular Polysaccharide Enables V. vulnificus to Evade Phagocytosis by Blocking Host‐Bacteria Interactions.” mBio 16, no. 3. 10.1128/mbio.03838-24 PMC11898582 39950808

[mbo370343-bib-0042] Lydon, K. A. , T. Kinsey , C. Le , P. A. Gulig , and J. L. Jones . 2021. “Biochemical and Virulence Characterization of Vibrio vulnificus Isolates From Clinical and Environmental Sources.” Frontiers in Cellular and Infection Microbiology 11: 637019. 33718284 10.3389/fcimb.2021.637019 PMC7952748

[mbo370343-bib-0043] Mala, W. , C. Chomvarin , M. Alam , S. M. Rashed , K. Faksri , and S. Angkititrakul . 2014. “Molecular Analysis of Vibrio Vulnificus Isolated From Cockles and Patients in Thailand.” Southeast Asian Journal of Tropical Medicine and Public Health 45, no. 1: 103–112. 24964659

[mbo370343-bib-0044] Matsuoka, Y. , Y. Nakayama , T. Yamada , et al. 2013. “Accurate Diagnosis and Treatment of Vibrio Vulnificus Infection: A Retrospective Study of 12 Cases.” Brazilian Journal of Infectious Diseases 17, no. 1: 7–12. 10.1016/j.bjid.2012.07.017 PMC9427351 23332442

[mbo370343-bib-0045] Mcgowan, D. , A. Kermani , and J. Sheagren . 2024. “Investigating and Summarizing Information Resources Related to the Clinical Presentation and Diagnosis of Cutaneous Manifestations of Infectious Diseases in Patients With Skin of Color.” Open Forum Infectious Diseases 11, no. 2: ofad692. 38390461 10.1093/ofid/ofad692 PMC10883730

[mbo370343-bib-0046] Mifflin, L. , D. Ofengeim , and J. Yuan . 2020. “Receptor‐Interacting Protein Kinase 1 (RIPK1) as a Therapeutic Target.” Nature Reviews Drug Discovery 19, no. 8: 553–571. 32669658 10.1038/s41573-020-0071-y PMC7362612

[mbo370343-bib-0047] Morgado, M. E. , K. D. Brumfield , C. Mitchell , and M. M. Boyle . 2024. “Increased Incidence of Vibriosis in Maryland, USA, 2006‐2019.” Environmental Research 244: 117940. 38101724 10.1016/j.envres.2023.117940 PMC10922380

[mbo370343-bib-0048] Okai, N. , K. Miyamoto , K. Tomoo , et al. 2020. “VuuB and IutB Reduce Ferric‐Vulnibactin in Vibrio vulnificus M2799.” BioMetals 33, no. 4–5: 187–200. 32681432 10.1007/s10534-020-00241-5

[mbo370343-bib-0049] Pajuelo, D. , C. Hernández‐Cabanyero , E. Sanjuan , et al. 2016. “Iron and Fur in the Life Cycle of the Zoonotic Pathogen.” Environmental Microbiology 18, no. 11: 4005–4022. 27348505 10.1111/1462-2920.13424

[mbo370343-bib-0050] Pérez‐Reytor, D. , V. Jaña , L. Pavez , P. Navarrete , and K. García . 2018. “Accessory Toxins of Vibrio Pathogens and Their Role in Epithelial Disruption During Infection.” Frontiers in Microbiology 9: 2248. 30294318 10.3389/fmicb.2018.02248 PMC6158335

[mbo370343-bib-0051] Qin, K. , K. Fu , J. Liu , C. Wu , Y. Wang , and L. Zhou . 2019. “Vibrio vulnificus Cytolysin Induces Inflammatory Responses in RAW264.7 Macrophages Through Calcium Signaling and Causes Inflammation In Vivo.” Microbial Pathogenesis 137: 103789. 31605759 10.1016/j.micpath.2019.103789

[mbo370343-bib-0052] Qin, K. W. , J. F. Liu , C. L. Wu , C. Zhang , and L. J. Zhou . 2023. “Resveratrol Prevents Vibrio Vulnificus‐Induced Sepsis by Attenuating Necroptosis.” Biomedical and environmental sciences: BES 36, no. 2: 135–145. 36861192 10.3967/bes2023.017

[mbo370343-bib-0053] Staley, C. , E. Chase , and V. J. Harwood . 2013. “Detection and Differentiation of Vibrio Vulnificus Andv. Sinaloensisin Water and Oysters of a Gulf of mexico Estuary.” Environmental Microbiology 15, no. 2: 623–633. 23240813 10.1111/1462-2920.12045

[mbo370343-bib-0054] Swartzendruber, J. A. , R. M. Del Toro , R. Incrocci , et al. 2022. “Lipopolysaccharide From the Cyanobacterium Geitlerinema Sp. Induces Neutrophil Infiltration and Lung Inflammation.” Toxins 14, no. 4: 267. 35448876 10.3390/toxins14040267 PMC9024439

[mbo370343-bib-0055] Trinh, S. A. , H. E. Gavin , and K. J. F. Satchell . 2017. “Efficacy of Ceftriaxone, Cefepime, Doxycycline, Ciprofloxacin, and Combination Therapy for Vibrio vulnificus Foodborne Septicemia.” Antimicrobial Agents and Chemotherapy 61, no. 12: e01106‐17. 10.1128/AAC.01106-17 PMC5700324 28971862

[mbo370343-bib-0056] Wang, D. , Q. Zheng , Q. Lv , et al. 2021. “Assessment of Seawater Bacterial Infection in Rabbit Tibia by Illumina Miseq Sequencing and Bacterial Culture.” Journal of Orthopaedic Surgery and Research 16, no. 1: 463. 34289854 10.1186/s13018-021-02553-9 PMC8293552

[mbo370343-bib-0057] Williams, T. C. , E. R. Blackman , S. S. Morrison , C. J. Gibas , and J. D. Oliver . 2014a. “Transcriptome Sequencing Reveals the Virulence and Environmental Genetic Programs of Exposed to Host and Estuarine Conditions.” PLoS ONE 9, no. 12: e114376. 25489854 10.1371/journal.pone.0114376 PMC4260858

[mbo370343-bib-0058] Williams, T. C. , M. Ayrapetyan , and J. D. Oliver . 2014b. “Implications of Chitin Attachment for the Environmental Persistence and Clinical Nature of the Human Pathogen.” Applied and Environmental Microbiology 80, no. 5: 1580–1587. 24362430 10.1128/AEM.03811-13 PMC3957613

[mbo370343-bib-0059] Wu, Z. , Y. Wu , H. Gao , et al. 2022. “Identification and Whole‐Genome Sequencing Analysis of Strains Causing Pearl Gentian Grouper Disease in China.” BMC Microbiology 22, no. 1: 200. 35974308 10.1186/s12866-022-02610-1 PMC9380395

[mbo370343-bib-0060] Xu, Y. , C. Liang , W. Zhang , et al. 2023. “Profiling of the Chemical Space on the Phenyl Group of Substituted Benzothiazole RIPK3 Inhibitors.” Bioorganic Chemistry 131: 106339. 36599218 10.1016/j.bioorg.2022.106339

[mbo370343-bib-0061] Yamazaki, K. , T. Kashimoto , T. Kado , K. Yoshioka , and S. Ueno . 2022. “Increased Vascular Permeability Due to Spread and Invasion of Vibrio vulnificus in the Wound Infection Exacerbates Potentially Fatal Necrotizing Disease.” Frontiers in Microbiology 13: 849600. 35350614 10.3389/fmicb.2022.849600 PMC8957983

[mbo370343-bib-0062] Yuan, Y. , Z. H. Feng , and J. L. Wang . 2020. “Vibrio Vulnificus Hemolysin: Biological Activity, Regulation of vvhA Expression, and Role in Pathogenesis.” Frontiers in Immunology 11: 599439. 33193453 10.3389/fimmu.2020.599439 PMC7644469

[mbo370343-bib-0063] Zhang, T. , S. Ji , M. Zhang , et al. 2024. “Effect of Capsular Polysaccharide Phase Variation on Biofilm Formation, Motility and Gene Expression In.” Gut Pathogens 16, no. 1: 40. 39075606 10.1186/s13099-024-00620-0 PMC11287873

[mbo370343-bib-0064] Zhao, J. , A. Sun , P. Ruan , X. Zhao , M. Lu , and J. Yan . 2009. “Vibrio vulnificus Cytolysin Induces Apoptosis in HUVEC, SGC‐7901 and SMMC‐7721 Cells via caspase‐9/3‐Dependent Pathway.” Microbial Pathogenesis 46, no. 4: 194–200. 19167479 10.1016/j.micpath.2008.12.005

